# Measuring the primary cilium length: improved method for unbiased high-throughput analysis

**DOI:** 10.1186/s13630-016-0028-2

**Published:** 2016-02-11

**Authors:** Anneloes Dummer, Christian Poelma, Marco C. DeRuiter, Marie-José T. H. Goumans, Beerend P. Hierck

**Affiliations:** Department Anatomy and Embryology, Leiden University Medical Center, Leiden, The Netherlands; Laboratory for Aero & Hydrodynamics, Delft University of Technology, Delft, The Netherlands; Department Molecular Cell Biology, Leiden University Medical Center, Leiden, The Netherlands

**Keywords:** Primary cilia, Cilium length, Measurements, Methods

## Abstract

**Background:**

Primary cilia are cellular protrusions involved in mechanic and chemical sensing on almost all cells of our body. Important signaling pathways, including Hedgehog, TGFβ, and Ca^2+^, are linked to cilia and/or cilia function. Cilia can vary in length, which has functional implications. To measure these lengths correctly, a standardized method with high reliability and throughput is required. To date, methods for length measurements in cultured cells after fluorescent staining for ciliary components are error prone with a possible human selection bias, primarily caused by the orientation of cilia with respect of the imaging plane. In tissue sections, accurate measurements become an even larger challenge due to additional random sectioning plane. Cilia can be reconstructed in 3D and measured one by one, but this is a labor-intensive procedure. Therefore, we developed a new, high-throughput method with less selection bias.

**Results:**

To identify the optimal type of measurement of straight and relatively short cilia, three methods were compared. The first method is based on maximum intensity projection (MIP), the second method is based on the Pythagorean theorem (PyT), and the third is based on 3D alternative angled slicing (DAAS). We investigated whether cilia visible in the plane of focus (‘flat cilia’), and the ones that are angled with respect to the plane of focus are represented differently among the various methods. To test the agreement between the methods, intraclass correlations are calculated. To measure flat cilia, MIP and DAAS provided representative results, with the MIP method allowing for higher throughput. However, when measuring the angled cilia with MIP, the actual cilium length is overtly underestimated. DAAS and PyT are exchangeable methods for length measurements of the angled cilia, while PyT exhibits higher throughput and is therefore the preferred method for measuring the length of an angled cilium.

**Conclusion:**

PyT is a universal measuring method to measure straight cilia, without selection bias. MIP provides similar results for flat cilia, but underestimates the length of angled cilia. In addition, PyT facilitates high-throughput length measurements. Manual tracking or reconstruction will be the method of choice to measure irregularly shaped cilia.

**Electronic supplementary material:**

The online version of this article (doi:10.1186/s13630-016-0028-2) contains supplementary material, which is available to authorized users.

## Background

Endothelial primary cilia are present in areas of disturbed blood flow [1] and are demonstrated to be involved in cell signaling processes involving, for example, Ca^2+^, TGFβ, Hedgehog (Hh), Wnt, cAMP/mTOR, and PDGFRα [2–8]. Along the axoneme, intraflagellar transport (IFT) regulates the availability of proteins for signal transduction, such as members of the Hh signaling cascade [9] and regulates the availability of building blocks for the axoneme to establish the cilium [10]. One of the many important functions of the primary cilium in endothelial cells is mechano-sensing at the liquid–tissue interface by bending under influence of flow [11], resulting in mechanistic stress on the cytoskeleton [12]. Such fluid flow sensing capacities have been described in, for example, the vasculature [13], kidney [14], cartilage [15], and bone tissue [16], to be necessary for correct functioning of the cells and concomitant tissue or organ hemostasis although cilium length can vary between cell types (Table 1). In addition to mechano-sensing via the microtubular network and Ca^2+^ influx, several proteins are located in the cilium for direct signaling, via, for example, the Hh signaling pathway [17]. Activating these pathways leads to altered gene expression and adapted reactions of the cell.

**Table 1:** Among various cell types, the length of the cilium can vary between 1 and 9 μm

Cell type	Primary cilium length	Reference
Vascular endothelial cells	1–5 μm	[1]
Kidney epithelial cells	5–6 μm	[23]
Neurons	4–9 μm	[37, 38]
Osteoblasts	3–4 μm	[16, 39]
Chondrocytes	2 μm	[15]

These characteristics are not unique for endothelial cilia. In fact, although ciliary lengths and shapes may differ, ciliary functions appear much conserved among cell types [18]. Adaptations of cilium length might affect various processes. First of all, a longer cilium will increase the torque, which is the cross product of the lever-arm vector and the force vector, leading to a renewed force equilibrium. The longer the cilium is, the less force is needed to bend the cilium and, for example, activate the Ca^2+^ influx. As proposed by Resnick et al., the longer the cilium, the more sensitive the cell becomes to flow changes [19].

Secondly, concomitant with the length increase, the volume of the cilium will increase leading to potential concentration adaptations of ions, proteins, and signaling molecules, and resulting biological responses. Thirdly, IFT will take longer in extended cilia [20]. As a result, it takes longer for proteins to reach the signaling hotspot at the tip of the cilia [9]. In fact, IFT velocity is one of the processes which regulate cilium length [21–23].

Besides intracellular signaling effects, the cell membrane also increases in surface area due to cilium elongation. Since the composition of the ciliary membrane is highly regulated and contains a distinct population of receptors [24], increased membrane area might affect the number of available receptors for signaling. Altogether, differences in cilium length might regulate and fine-tune signaling in the cilium and in the cell.

The length of cilia can change in conditions such as injury [25] and inflammation [15]. When studying the effects of cellular processes on cilium length, or vice versa, it is important to measure cilia in an accurate way.

**Fig. 1:**
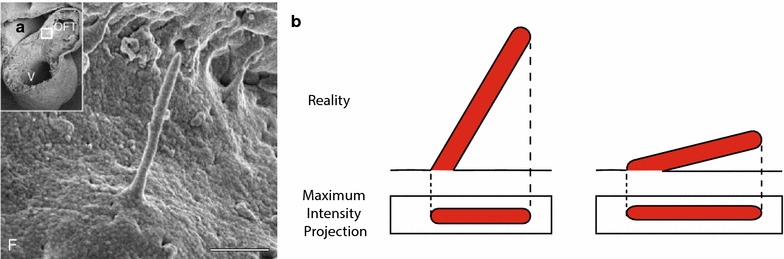
Visual appearance of a primary cilium. **a**. Scanning Electron Microscopy image in the developing heart showed a continuous slope of the endothelial primary cilium. *Scale bar* is 1 μm. Re-used with permission and adapted from van der Heiden et al., 2006 [33]. **b** Schematic overview of the difference between real length of the cilium and visibility on a maximum intensity projection

Due to the biological feature that cilia are protruding organelles and extend into the extracellular space to function as a sensor, cilia are considered to have a 3D orientation. This assumption is supported by electron scanning microscopy (Fig. 1) where cilia are visible in an angled way with respect to the cell surface.

Fluorescent confocal imaging is the most common and high-throughput analysis technique for the determination of cilium length. However, the cilia present in random orientation within the microscopic plane. For example, in cell culture the majority of cilia protrude more or less perpendicular to the cell surface, and might not be visible in one plane of focus. These cilia will be called *angled cilia* here. However, other cilia are lying in the plane of focus, possibly tilted during the process of sample preparation. These cilia will be called *flat cilia*. These definitions also apply for cilia in tissue sections, where cilia extend in multiple directions due to anatomical tissue organization. Many reports appear to focus on the flat cilia or on a projection of cilia, which might result in an underestimation of cilium length (Fig. 1a). With the assumption that flat and angled cilia represent the same population of cilia, selecting only the flat cilia and measuring length with the maximum intensity projection (MIP) will be sufficient. Like when all cilia are visible as flat cilia as e.g. in retinal pigment epithelial (RPE) cells. However, this assumption inherits the risk of selection bias toward the flat cilia, which may or may not represent a different population of cilia. In this report, we analyze various measurement techniques for measuring straight cilia and discuss their pros and cons.

## Methods

### Cell culture

Cultured ciliated mouse embryonic endothelial cells (MECs) [26] were used in this study. Cells were cultured as previously described [27].

For forskolin treatment [23], cells were grown on 10-mm coverslips until confluence to stimulate maximal ciliogenesis [27] upon which the medium was supplemented with 100 nM forskolin (Sigma-Aldrich), in dimethylsulfoxide (DMSO, Sigma-Aldrich). Forskolin was used to stimulate ciliary elongation [23]. DMSO was also used as sham control. Cells were fixed after 24 h with 4 % paraformaldehyde (Merck) in 0.1 mol/L phosphate buffer (pH7.4) for 10 min at room temperature. All experiments were repeated three times. Representative samples are shown in the Results section.

### Immunofluorescence

Fixed cells were permeabilized by PBS with 0.05 % Tween 20 (Merck) and incubated with an antibody against acetylated-α-tubulin (6-11B-1, 1:2000, Sigma-Aldrich) for 3 h at 37 °C. Subsequently, the cells were incubated with Cy3-goat anti-mouse antibody (1:500, Vector Laboratories) for 30 min at room temperature followed by a DAPI (1:1000, Molecular probes) staining for 5 min. Cells were mounted in Prolong Gold (Molecular probes). Confocal images were taken with a Leica SP5 confocal Microscope (Leica) with a 405 nm diode and 561 nm Helium–Neon laser and the 63x oil objective. Z-stacks are used for the MIP, PyT, and DAAS methods as described in the results section.

Staining with an antibody against Arl13b (17711-1-AP, 1:500, Proteintech) was used to confirm cilia staining (Additional file 1: Figure S1a).

### Different cilia populations

Flat cilia were defined by the appearance of their entire length in a maximum of four Z-slices, according to previous descriptions [28–31]. In contrast, the angled cilia were visible in more than four slices and changed in visible shape while moving through the Z-stack (Additional file 2: Movie S1).

### The three methods

**Fig. 2:**
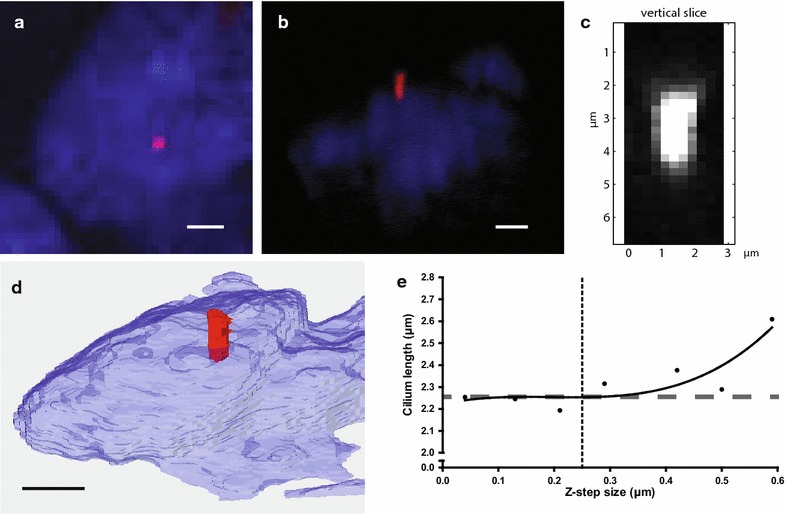
Visual appearance of the same cilium with the different methods. Visual appearance of the same angled cilium with the MIP method (**a**), PyT method (**b**) (see also Additional file 4: Movie S2), DAAS method (**c**), and a reconstruction with Amira (**d**). *Scale bars* are 3 μm. **e**. Length of a cilium, measured with different sizes of the z-slices using the PyT method. *Dotted line* indicates z-slice size of 0.25 µm, and *dashed line* indicates the cilium length (2.26 μm) measured by 3D reconstruction using Amira

The cilia were analyzed using three different methods based on confocal Z-stacks. A visualization of the three methods is shown in Fig. 2a–c. The first method is based on the MIP (MIP method, Fig. 2a), the second method is derived from the Pythagorean theorem (PyT method, Fig. 2b, Additional file 2: Movie S2), and the last method is based on a 3D reconstruction with alternative angled slicing through the Z-stack (DAAS method, Fig. 2c).

Cilium length was measured with ImageJ 1.48v (MIP and PyT, http://imagej.nih.gov/ij) or Matlab (DAAS, R2014b, The Mathworks). To limit measurement errors, for each cilium the average of three measurements was used for statistical analysis. Moreover, inter-observer variation was covered by repeating measurements by an independent observer.

For 3D reconstruction of cilia, Amira^®^ software package version 5.6 was used (Template Graphics Software; Visage Imaging, San Diego, California, USA) (Fig. 2d).

Staining with an antibody against Arl13b (17711-1-AP, 1:500, Proteintech) confirmed cilia length measurements as seen by acetylated-α-tubulin (Additional file 3: Figure S1b).

Staining and length measurements were also confirmed in human primary microvascular endothelial cells (data not shown).

### Statistics

To perform the statistical analyses described in the Results section, SPSS Statistics 20 (IBM) was used.

## Results

### Z-slices

The optimal distance between the Z-slices was determined by comparing Z-stacks with different step sizes for reconstruction of the same cilium. Steps ranging from 0.04 to 0.40 µm did not show any differences in visual appearance of the cilia and the length of the cilia (Fig. 2b). Step sizes larger than 0.40 µm showed more variance in cilium length and were considered not reliable (Fig. 2e). To achieve maximum accuracy with the highest throughput in confocal imaging, the step size was established at 0.25 µm which is in agreement with McGlashan et al. [31].

### Method PyT, using the Pythagorean theorem

**Fig. 3:**
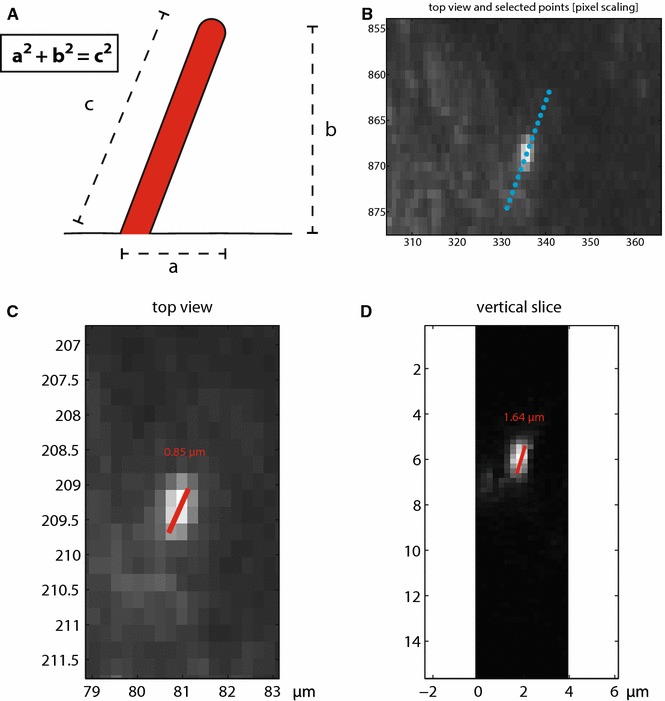
The PyT and the DAAS methods. **a**. PyT method. With the assumption of a continuous slope, the Pythagorean theorem can be applied to calculate the length of a cilium. **b**–**d**. DAAS method. After selecting a cilium and the cross section line (**b**), the program gives a top view (**c**) and the vertical slice of the selected plane (**d**). Measurements are performed in Matlab. Cilia are measured on grayscales from 55 to 255

With the knowledge of the length of two sides of a right triangle, the method facilitates the calculation of the third side by the formula* a*^2^ + *b*^2^ = *c*^2^. This can also be applied to a cilium (Fig. 3a). For measuring the actual cilium length *c*, the length of the cilium on the MIP is used as *a*, and the number and thickness of Z-slices will provide *b*.

### Method DAAS, 3D alternative angled splicing

In this method, the operator is first presented with a MIP representation of the confocal image stack. In this ‘top view‘ (Fig. 3b), two points are selected that set up a vector aligned with a particular cilium (i.e., a point at the beginning and end of the cilium). This line is then used to create a slicing plane defined by the selected vector (in the x–y plane) and the z-axis (i.e., z-stack direction). A new image is created by bilinear interpolation of the 3D stack. The interpolation locations are on a regular grid using the same resolution as the original data, encompassing the cilium region. This interpolation process provides a ‘side view’ of the cilium (Fig. 3d). Two or more points can now be selected along the cilium to determine its length. Once completed, the measured cilium is labeled in the MIP view and the process can be repeated until all cilia in the data set are measured. This approach (including graphical user interface) was implemented in Matlab (R2014b, The Mathworks).

### Statistics

#### Comparing the flat and angled cilia

When considering cilia in a confluent monolayer of cells, it is yet unclear whether the flat cilia and the angled cilia represent the same population of cilia. To define if all cilia can be measured in the same way, a possible length bias between the flat and angled cilia should be excluded. To test this, the two populations were compared within all methods. The distribution of both populations is circa 50/50. The differences of the average lengths were tested with an independent student’s *T* test.

**Fig. 4:**
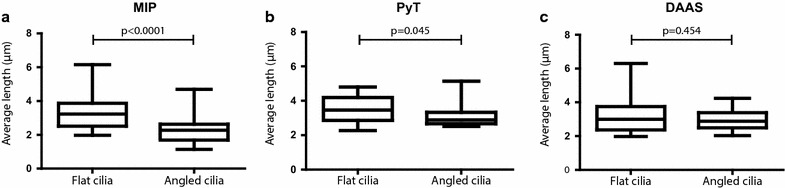
Cilium length between the different populations measured in three different methods. **a** Measurements with the MIP method. **b** Measurements with the PyT method. **c** Measurements with the DAAS method. In all methods, the same sample of cilia is used. Boxplot contains the 25–75 percentile, and the *whiskers* represent the minimum and maximum of the measurements. *n* = 31 for both the flat and angled cilia

First of all, when measured with MIP (Fig. 4a), the angled cilia have an average length of 2.30 ± 0.74 μm and the flat cilia of 3.28 ± 0.92 μm, which represents a significant difference (*p* < 0.0001). When the same cilia are measured with the PyT method (Fig. 4b), the angled cilia have an average length of 3.10 ± 0.61 μm and the flat cilia of 3.46 ± 0.74 μm (*p* = 0.045). When the cilia are measured with DAAS (Fig. 4c), the angled cilia have an average length of 2.95 ± 0.60 μm and the flat cilia of 3.10 ± 0.92 μm (*p* = 0.454) suggesting no structural difference in length.

#### Comparing different methods

**Fig. 5:**
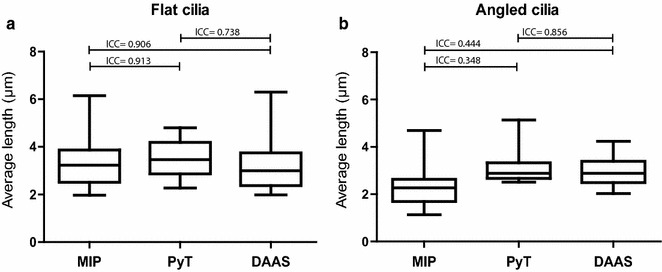
Comparison of the flat and angled cilia. **a** Comparing the different methods for the population of flat cilia. **b** Comparing the different methods for the population of angled cilia. ICC = intraclass correlation coefficient. Interpretation of the ICC according to Landis and Koch, 1977: kappa value of <0.2 = slight agreement; >0.2–0.4 = fair; >0.4–0.6 = moderate; >0.6–0.8 = substantial; >0.8 = almost perfect agreement. *n* = 31 for both the flat and angled cilia

To test the agreement between the different methods, intraclass correlation coefficients (ICCs) are calculated (Fig. 5) and Bland–Altman plots are created (Additional file 3: Figure S2).

The output of the intraclass correlation is a kappa value which reflects a possible bias, and can be interpreted according to Landis and Koch [32]: The higher the kappa value, the more comparable the methods are (ranging from <0.2 as poorly and >0.8 as almost perfectly in agreement). As shown in Fig. 5a, the flat cilia have a high intraclass correlation between all methods. However, it should be noted that although the ICC between PyT and DAAS is substantial (ICC = 0.74), it is slightly lower than the ICC between MIP and DAAS (ICC = 0.91). This suggests that measurements of the flat cilia are more accurate with MIP than with PyT.

On the other hand, the angled cilia show a low correlation when they are measured with MIP compared to either PyT or DAAS (Fig. 5b). The correlation between PyT and DAAS is high for the angled cilia, therefore making the methods exchangeable for the angled cilia population.

To visualize the variation between the various measurement techniques of the same sample, Bland-Altman plots can be used [33]. In this plot (Additional file 3: Figure S2), the differences of the measurements are plotted (y-axis) against the average lengths on the x-axis. These plots show the 95 % agreement interval of the measurements of the same cilium among the methods. The average difference in agreement directly provides the overall bias between the two methods. Whether the width of the 95 % agreement interval and bias are acceptable relies on the topic. By this, the plots have improved interpretation of the data from a biological viewpoint beyond just looking at significant changes.

When the different methods MIP, PyT, and DAAS are plotted against each other, the distribution of differences between the methods becomes visible.

Comparing MIP with DAAS for the flat cilia, the distribution cloud is largely spherical, and the average difference is 0.19 ± 0.54 µm. However, when comparing MIP with PyT, the differences are on average −0.29 ± 0.29 µm, indicating less variation between the methods since the 95 % agreement interval is smaller, but a larger bias as seen by the average difference. Comparing the flat cilia between PyT and DAAS, the intraclass correlation is lower (ICC = 0.74) and the differences are on average 0.48 ± 0.58 µm. This indicates a bias of 0.48 µm difference between the measurement methods when comparing the flat cilia. Taken together, these data indicate that MIP and DAAS show the best agreement on measuring the flat cilia.

When the angled cilia are analyzed, the comparison between MIP and DAAS shows a low intraclass correlation (0.44) and concurrent low average of −0.65 ± 0.90 µm. When MIP is compared to PyT, the average of differences is −0.80 ± 0.82 µm. For both cases, the average differences indicate a large bias and the large spreading of the differences indicates low agreement when comparing MIP to either DAAS or PyT for the angled cilia. When comparing PyT and DAAS for the angled cilia, the average difference is 0.16 ± 0.57 µm indicating a smaller bias and a smaller spreading of the agreement interval. Together, these data indicate that PyT and DAAS show the best agreement to measure angled cilia.

Altogether, considering both the intraclass correlation coefficient (ICC) and the Bland–Altman analyses, the measurements of the flat cilia have more agreement when measured with MIP and DAAS. The measurements of the angled cilia show the highest agreement when measured with PyT and DAAS.

## Discussion

To date, there is no consensus on the best way to measure cilium length in cultured cells or histological specimens. Many cilia will not be aligned in the imaging plane, in histological specimens even less because of additional variations in sectioning plane, and are therefore difficult to visualize within one plane. Here we show that endothelial cilia are positive for acetylated-α-tubulin (axonemal staining) and Arl13b (membrane staining), and that the use of both markers results in identical length measurements. However, one should realize that some cell types may have long and irregularly shaped cilia. In this case, manual tracking, with the risk of selection bias, remains the only method to measure their lengths. According to Saggesse et al., the most accurate method is to reconstruct each cilium, but this is time consuming and requires specific software and concomitant expertise for image processing [30]. Many reports focus only on the flat cilia, using the MIP method. Although it is assumed that the flat and angled cilia are comparable, only considering a small subset of cilia might create a bias. Moreover, when cilia do not align properly to the plane of focus but are still defined as flat cilia, the measured length is an underestimation (Fig. 1a). In addition to reliable length measurements, high throughput is important to increase the amount of cilia included in analyses and provide less risks for bias.

The PyT method provides the ability to measure many or even all cilia in one sample, thereby preventing any selection bias. Moreover, by calculating cilium length using PyT instead of reconstructing every cilium, a higher throughput can be generated. When using PyT method, it has to be assumed that the slope of the cilia is continuous along the complete length of the cilium. A continuous slope of endothelial primary cilia has been shown already by scanning electron microscopy (Fig. 1a) [34]. Moreover, microtubules are considered the most rigid structures of the cellular cytoskeleton [35] and form the basic structure of the primary cilia [18]. Furthermore, the axoneme is constructed of 9 microtubule doublets even adding to the rigidity of the cilium. Although cilia sometimes appear irregular, this might be due to preparation artifacts.

Using method DAAS provides a view on the cilium in the three-dimensional space. It is an accurate but time-consuming way to measure the cilium length and relies on specific software.

To exclude a structural bias between the flat and angled cilia, we analyzed the two populations in our endothelial specimens. Using the DAAS method, the angled cilia do not have a significantly different length compared to flat cilia. Comparing the two populations in the MIP method shows a significant difference, indicating that MIP is not a good measurement instrument to determine the angled cilia length. When the two populations are compared in the PyT method, the *p* value of 0.045 stresses the importance of biological interpretation of the data, as was demonstrated with the intraclass correlation and Bland–Altman analyses. Although these show that there is still a small bias comparing the methods, it is questionable whether a difference of 0.16–0.19 µm is biologically relevant. Although in the context of a cilium length of 2 µm it represents a possible 8–10 % bias, it completely lies within the technical range of errors which is a maximum of 0.3 µm among three repeated measurements. Moreover, the average cilia length within one sample has a standard deviation of 0.60–0.92 µm, exceeding the bias.

When cilia are straight and it is demonstrated that the flat and angled cilia represent the same cilia population, MIP can be sufficient to determine ciliary length. However, implementing the PyT method to include all cilia present will avoid any possible selection bias. Although in this study no length differences in endothelial flat and angled cilia are demonstrated, this should be confirmed for other cell types.

If cilia present as irregularly shaped structures, it is important to consider that this might be a natural phenomenon or be an artifact of the fixation and/or staining procedure. In either case, the PyT method will not suffice to measure ciliary length. However, if these cilia are completely visible as flat cilia or overlap, the MIP method, in combination with manual tracking or 3D reconstruction, will be necessary for length measurements, even if the cilia are up to 100 μm [36]. In this paper, we show that the PyT method is comparable to DAAS to measure the length of primary cilia and therefore are exchangeable. Especially, for the most common straight cilia in either tissue sections or cell culture, the PyT method is an improvement compared to MIP.

Automation of 3D reconstruction could improve throughput, but appears to be complex in reality. Perhaps, future improvements in imaging and computer techniques will allow the use of this technique in high-throughput analyses. This also applies when considering scanning electron microscopy to measure cilium length. Lack of throughput and shrinkage due to dehydration render this a visualization tool rather than a measurement tool.

## Conclusion

The current method of measuring only flat cilia on a maximum intensity projection may give a good representation of the cilium length. However, measuring all cilia within one image using the PyT method, including all angled cilia, will give an improved representation of the complete cilia population. The flat cilia can be properly measured in the MIP method which equals the PyT method without Z-depth, while the angled cilia should be measured in a more 3D way where method PyT and method DAAS are exchangeable. However, method PyT can exhibit higher throughput than DAAS and therefore provides the possibility of measuring many or all cilia in the same sample giving a better representation of the complete cilia population. Consequently, human selection bias can be prevented in this way. Furthermore, it is very important to consider the interpretation of the statistics when looking at cilium lengths. Although statistics may give a significant difference, the biological context should always be considered.

In conclusion, the PyT method is a reliable approach to measure cilium length in a high-throughput manner and provides an improved generic tool to measure cilium length.

## Additional files

10.1186/s13630-016-0028-2 Confirmation cilium staining and length measurements.
10.1186/s13630-016-0028-2 The visualization of scrolling through the z-stack, showing an angled cilium. The cell is stained with acetylated-alpha-tubulin to visualize the cilium. Moving through the Z-stack, it is visible that the visible form of the cilium changes and moves, suggesting an angled cilium.
10.1186/s13630-016-0028-2 The Bland–Altman plots of the comparison between the different methods.
10.1186/s13630-016-0028-2 Scrolling through the z-stack of Fig. 2b, used for the calculation for the cilium length. The cell is stained with acetylated-alpha-tubulin to visualize the cilium. The amount of Z-stacks in which the cilium is visible is used for calculation cilium length in the PyT method.

## Abbreviations

3D: three-dimensional
DAAS: 3D alternative angled slicing
ICC: intraclass correlation coefficient
IFT: intraflagellar transport
MIP: maximum intensity projection
PyT: Pythagorean theorem method
