# Supplementary figures and images for: Measuring the primary cilium length: improved method for unbiased high-throughput analysis
Source: Cilia. 2016 Feb 11;5:7. doi: 10.1186/s13630-016-0028-2 (PMC4750300; doi:10.1186/s13630-016-0028-2)

Additional Figure 1

**A**

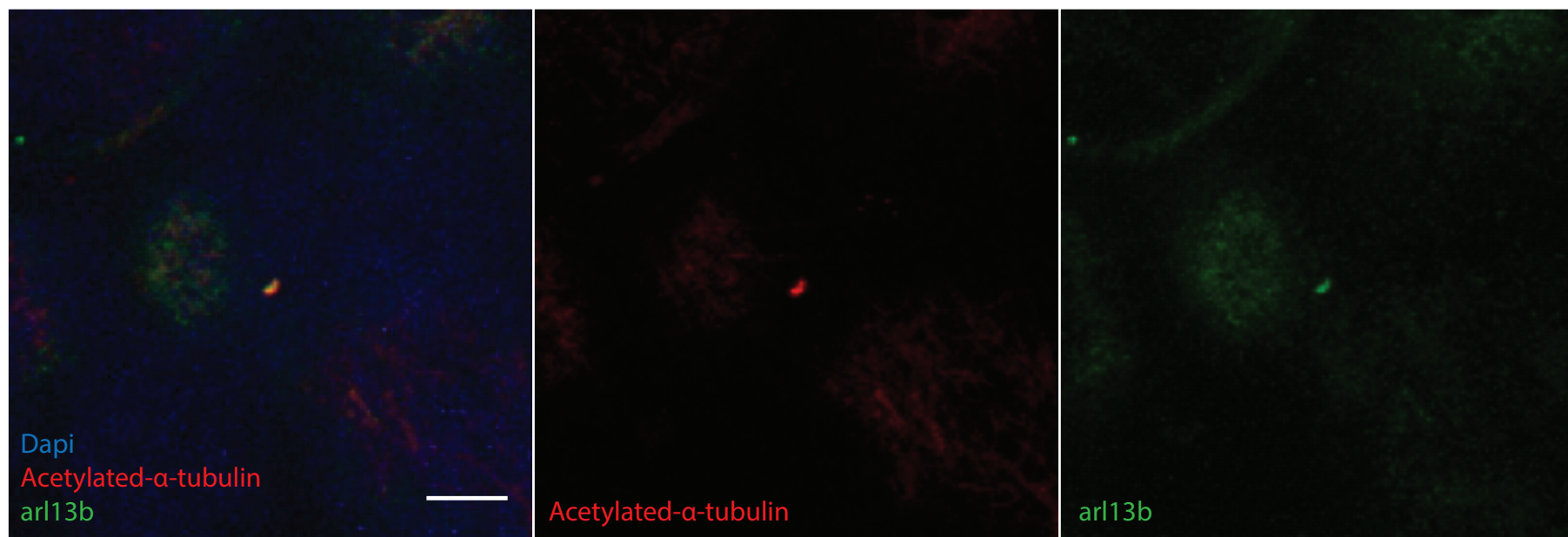

**B**

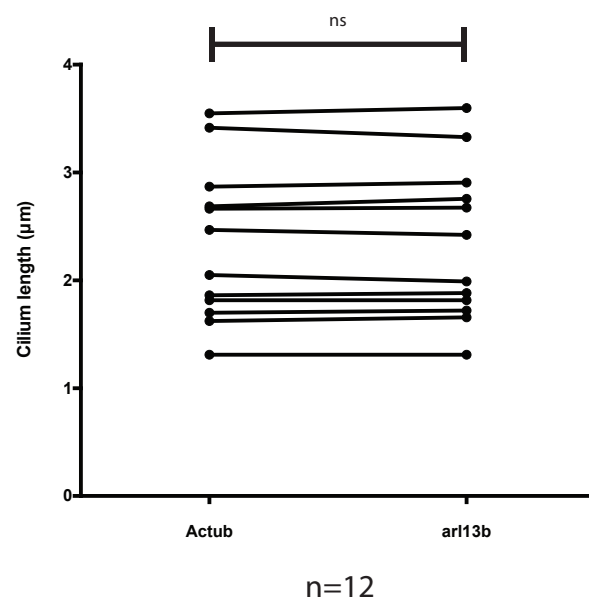

Supplement: Supplementary file 1 — 10.1186/s13630-016-0028-2 Confirmation cilium staining and length measurements. [file 13630_2016_28_MOESM1_ESM.pdf]

Additional Figure 2

Flat cilia

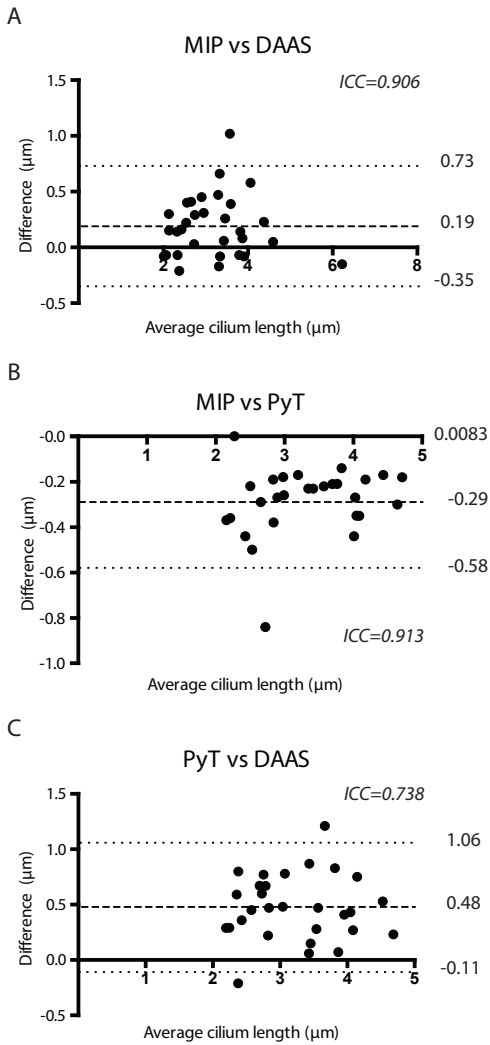

Angled cilia

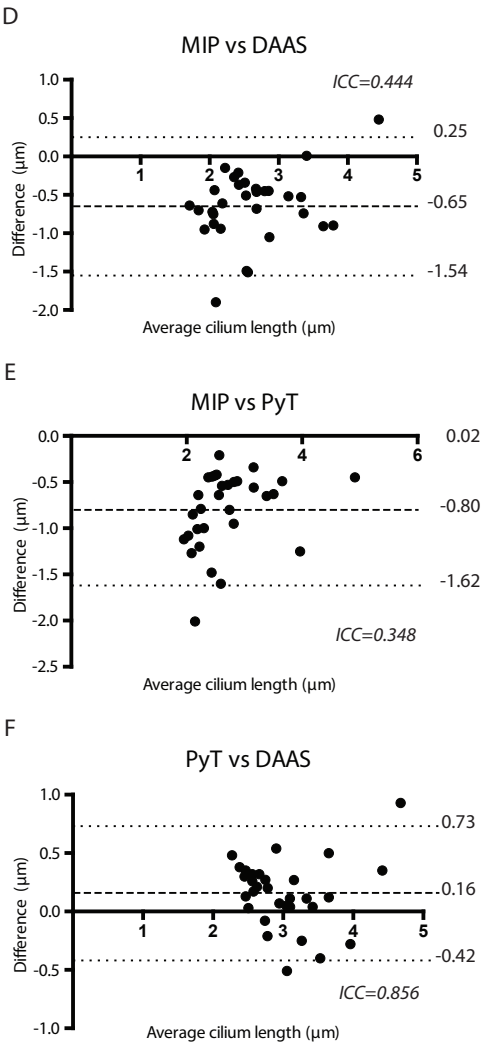

Supplement: Supplementary file 3 — 10.1186/s13630-016-0028-2 The Bland–Altman plots of the comparison between the different methods. [file 13630_2016_28_MOESM3_ESM.pdf]
